# Synthesis, Composition, and Properties of Partially Oxidized Graphite Oxides

**DOI:** 10.3390/ma12152367

**Published:** 2019-07-25

**Authors:** Michal Lojka, Boris Lochman, Ondřej Jankovský, Adéla Jiříčková, Zdeněk Sofer, David Sedmidubský

**Affiliations:** Department of Inorganic Chemistry, Faculty of Chemical Technology, University of Chemistry and Technology, Technická 5, 166 28 Prague 6, Czech Republic

**Keywords:** graphite oxide, modified Tour method, partial oxidation, graphene derivatives

## Abstract

The aim of this paper is to prepare and characterize partially-oxidized graphite oxide and consider if it is possible to affect the level of oxidation of particles by an adjustment of the oxidizing agent. Several samples were prepared, using different amounts of oxidizing agent. The samples were subsequently analyzed. The C/O ratio was evaluated from XPS, EDS, and EA. The amount and type of individual oxygen functionalities were characterized by XPS, Raman spectroscopy, and cyclic voltammetry. The structure was studied by SEM and XRD. Thermal stability was investigated by STA-MS in argon atmosphere. The results can be useful in order to design simple technology for graphite oxide synthesis with required oxygen content.

## 1. Introduction

Research on 2D carbon-based nanomaterials has become very intensive since the discovery and first successful isolation of graphene in 2004 [1,2]. Many different procedures of the preparation [3] and further functionalization of graphene have been reported [4]. The top-down approach is one of the possible methods for its mass production [5]. This method is based on graphite/graphene oxidation, followed by the subsequent reduction/exfoliation of graphite/graphene oxide. This is possible due to the increase in interlayer distance between individual carbon layers [6,7,8]. 

On the other hand, for the fabrication of precise monolayer graphene material, the bottom-up approach has to be used [9]. The most broadly used method is chemical vapor deposition (CVD), where small saturated carbohydrates, such as methane, are decomposed at a reachable temperature, around 900 °C, on a highly smoothed copper or nickel surface [10,11]. Another approach is the decomposition of silicon carbide (SiC), since it is directly prepared on the silicon wafer surface. Nevertheless, the fabrication of ultrapure SiC itself represents a technological issue and is not reachable in large-scale production [9]. Interestingly, a method for the precise patterning of graphite/graphene has been reported [12].

Since the first preparation of graphite oxide (GO) in the 19th century [13], several possible structures for this material have been reported [14,15,16,17,18,19]. Generally, graphite oxide is a lamellar compound consisting of carbon layers. These layers have been wrinkled during the oxidation process due to change in the hybridization of carbon atoms, with the simultaneous addition of oxygen-containing functionalities (hydroxyls, epoxides, ketones, carboxyls, etc.). Carboxyl functionalities are usually found at the edges of individual layers. Other oxygen functionalities, such as hydroxyls, epoxides, or ethers, used to be found on the surface of the layers [6]. Nevertheless, the exact structure of the prepared material is strongly influenced by the starting material used, oxidation process, and even purification process [6,20,21]. Compared to pristine graphite, the interlayer distance significantly increases during the oxidation, from 3.4 Å to 6–12 Å, based on the procedure [6,22,23]. The interlayer distance within individual graphene sheets is a crucial factor for subsequent exfoliation or further processing. Also, the presence of water molecules intercalated into the interlayer space is obvious and cannot be ignored when describing GO [24]. The C/O ratio is also very dependent on the method used and can vary significantly. Usually, a C/O ratio from 1.8 to 2.5 can be achieved in fully oxidized GO. Recently, Dimiev et al. reported that the C/O ratio does not change significantly with the addition of more than four equivalents of potassium permanganate compared to original graphite, and this addition is, thus, not necessary [24].

Since graphite is a very inert mineral, it can undergo oxidation only with very strong oxidation agents. There are several methods for graphite oxide production [13]. These methods are usually termed in literature according to their inventors. Graphite oxides can be prepared from graphite by electrochemical [25] or chemical approaches [26]. Chemical approaches are based on the oxidation of carbon-based precursors (graphite, graphene, nanographite, amorphous carbon, single- and multiwall carbon nanotubes). The structure of the resulting graphite oxide and the presence of specific oxygen functionalities is strongly affected by the procedure used, which is caused by a different mechanism of oxidation [27,28]. Basically, two common oxidizing agents are used to prepare graphite oxide: potassium permanganate [29] and potassium chlorate [30,31]. Procedures using chlorates lead to the synthesis of graphite oxide with mainly hydroxyl and epoxy functionalities, and the planar structure of the material is preserved, whereas procedures using permanganate provide a product with mostly carboxyl functionalities and the wrinkled structure is observed [32]. In the recently described method of graphite oxidation, which is sometimes termed the Tour method [33], graphite powder is oxidized in a mixture of sulfuric and phosphoric acid in the ratio 9:1, respectively, using potassium permanganate as an oxidizing agent. Phosphoric acid is crucial for stabilizing the reaction. The recommended ratio of graphite and potassium permanganate is 1:6. Another modification of this method has been reported, where the time of the heating can be reduced to up to one hour instead of the original 12 [34]. Interestingly, a procedure using potassium ferrate (VI) as an oxidizing agent has been reported due to its extraordinarily strong reduction potential [35]. However, in an acidic environment K_2_FeO_4_ is not stable, since it can oxidize water in aqueous solutions [36].

Graphite oxide is not only a precursor for graphene synthesis, but it is also itself a suitable material for membrane fabrication [37] or for various hybrid materials [38,39]. Graphene oxide is also an essential material for the synthesis of functionalized graphene (e.g., halogenated or hydrogenated graphene [32,40]). Graphite oxide prepared via permanganate oxidation can also undergo subsequent re-oxidation [41,42]. During this reaction, the typical graphene structure disappears, forming an “amorphous” structure called graphene acid. This re-oxidation process led mainly to the formation of carboxyl functionalities in the sample. Graphene acid has a great potential to remove heavy metals from waste water [43,44] or for the synthesis of highly selective membranes. 

As apparent from the previous literature overview, there are many papers dealing with graphite oxidation, however, only a few papers address the synthesis of partially-oxidized graphite oxides. The aim of this paper is to prepare and characterize partially-oxidized graphite oxide and consider whether it is possible to affect the structure and the level of oxidation by an adjustment of the oxidizing agent. 

## 2. Materials and Methods 

Pure graphite (2–15 μm, 99.9999 wt.%) was purchased from Alfa Aesar (Haverhill, MA, USA), while potassium permanganate (99.5 wt.%), hydrogen peroxide (30 wt.%), sulfuric acid (98 wt.%), phosphoric acid (85 wt.%), and ethanol were purchased from Penta (Prague, Czech Republic).

A mixture of phosphoric acid and sulfuric acid was formed, with a volume ratio 40:360 mL. The mixture was cooled to 0 °C. In the next step, graphite (3.0 g) and, subsequently, KMnO_4_ (0.2 g, 0.5 g, 3.0 g, or 6.0 g) were added. Then, the mixture was intensively stirred at 50 °C for 2 h [34]. After this time, the mixture was cooled to 20 °C. When the temperature dropped below 20 °C, the mixture was poured onto ice, followed by the addition of 20 mL of hydrogen peroxide to remove the remaining KMnO_4_ and MnO_2_. The mixture was then decanted five times and dried in a vacuum drier to obtain pure material. 

Samples were analyzed by a broad spectrum of analytic methods. These methods are described in detail in the Supplementary Materials. The morphology was investigated by Scanning Electron Microscopy, SEM (Tescan Lyra dual beam microscope, Brno, Czech Republic), structure was analyzed by X-Ray Diffraction, XRD (Bruker D8 Discoverer powder diffractometer, Karlsruhe, Germany) and Raman spectroscopy (Renishaw, Wotton under Edge, UK), chemical composition was studied by Energy Dispersive Spectroscopy, EDS (Oxford Instruments, Abingdon on Thames, UK), elemental analysis, EA (PE 2400 Series II CHNS/O Analyzer, Perkin Elmer, Waltham, MA, USA), and X-ray Photoelectron Specroscopy, XPS (ESCAProbeP spectrometer, Omicron Nanotechnology Ltd., East Grinstead, UK). The surface area was measured using a sorption analyzer BET (Coulter SA 3100, Backman Coulter, Brea, CA, USA). Thermal stability was analyzed by Simultaneous Thermal Analysis–Mass Spectroscopy, STA-MS (Setaram, Lyon, France) in an inert atmosphere. Moreover, electrochemical behavior was measured by cyclic voltammetry (potentiostat PGSTAT 204, Metroohm, Prague, Czech Republic).

## 3. Results and Discussion

In this work, samples of partially-oxidized graphite oxides (poGOs) were prepared using a modified Tour method, with a reduced amount of oxidizing agent. Samples were termed according to the amount of KMnO_4_ used, as poGO-0.2 g, poGO-0.5 g, poGO-3.0 g, and poGO-6.0 g. All samples were analyzed by SEM, EDS, EA, XPS, XRD, BET, Raman spectroscopy, STA-MS, and cyclic voltammetry.

**Figure 1:**
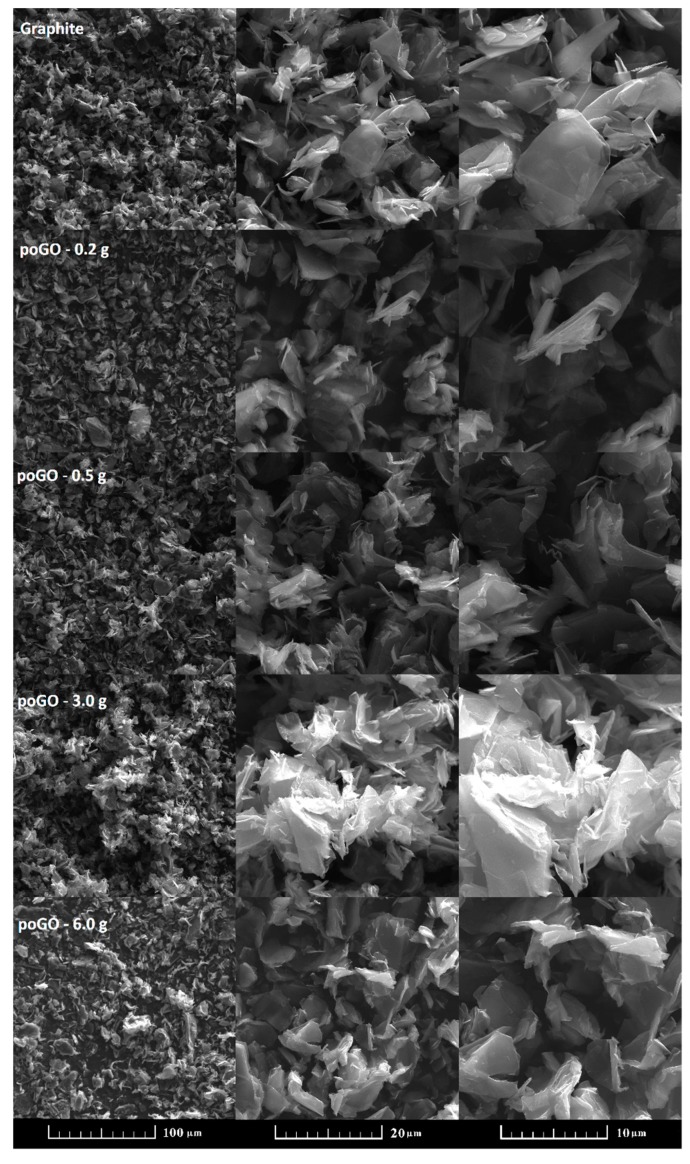
Micrographs of partially oxidized graphites poGOs.

The morphology of the prepared poGOs in comparison to the starting graphite powder was investigated by SEM (see Figure 1). It is obvious that the oxidation of the graphite did not significantly influence the structure, as the planar layered microstructure characteristic to graphite remained. EDS was measured simultaneously with SEM analysis. In the sample poGO-0.2 g, the chemical composition was 92.4 at.% carbon, 7.4 at.% oxygen, and 0.2 at.% sulfur. In the second sample, poGO-0.5 g, the oxygen content increased, the obtained chemical composition was 89.2 at.% carbon, 9.8 at.% oxygen, and 1.0 at.% sulfur. A similar tendency was also found for the remaining samples. In the sample poGO-3.0 g, the composition was 76.8 at.% carbon, 23.1 at.% oxygen, and 0.1 at.% sulfur, while for poGO-6.0 g it was 71.7 at.% carbon, 28.2 at.% oxygen, and 0.1 at.% sulfur. Potassium, manganese, and phosphorus were not found by SEM-EDS analysis. The obtained C/O ratios are compared in Table 1.

Chemical composition was also measured by EA. From the principle of this method, only nonmetal elements can be detected; on the other hand, hydrogen content can be measured. In the sample poGO-0.2 g, the chemical composition was 85.4 at.% carbon, 7.1 at.% oxygen, 6.7 at.% hydrogen, and 0.8 at.% sulfur. Sample poGO-0.5 g contained 78.6 at.% carbon, 9.1 at.% oxygen, 11.2 at.% hydrogen, and 1.2 at.% sulfur, while poGO-3.0 g contained 64.9 at.% carbon, 17.5 at.% oxygen, 16.2 at.% hydrogen, and 1.4 at.% sulfur. The last sample, poGO-6.0 g, had the highest content of oxygen and hydrogen, its chemical composition was 59.8 at.% carbon, 19.5 at.% oxygen, 19.9 at.% hydrogen, and 0.9 at.% sulfur. Sulfur impurities were also detected. The origin of sulfur is in the synthesis procedure of GOs. The obtained C/O ratios are again compared in Table 1.

**Figure 2:**
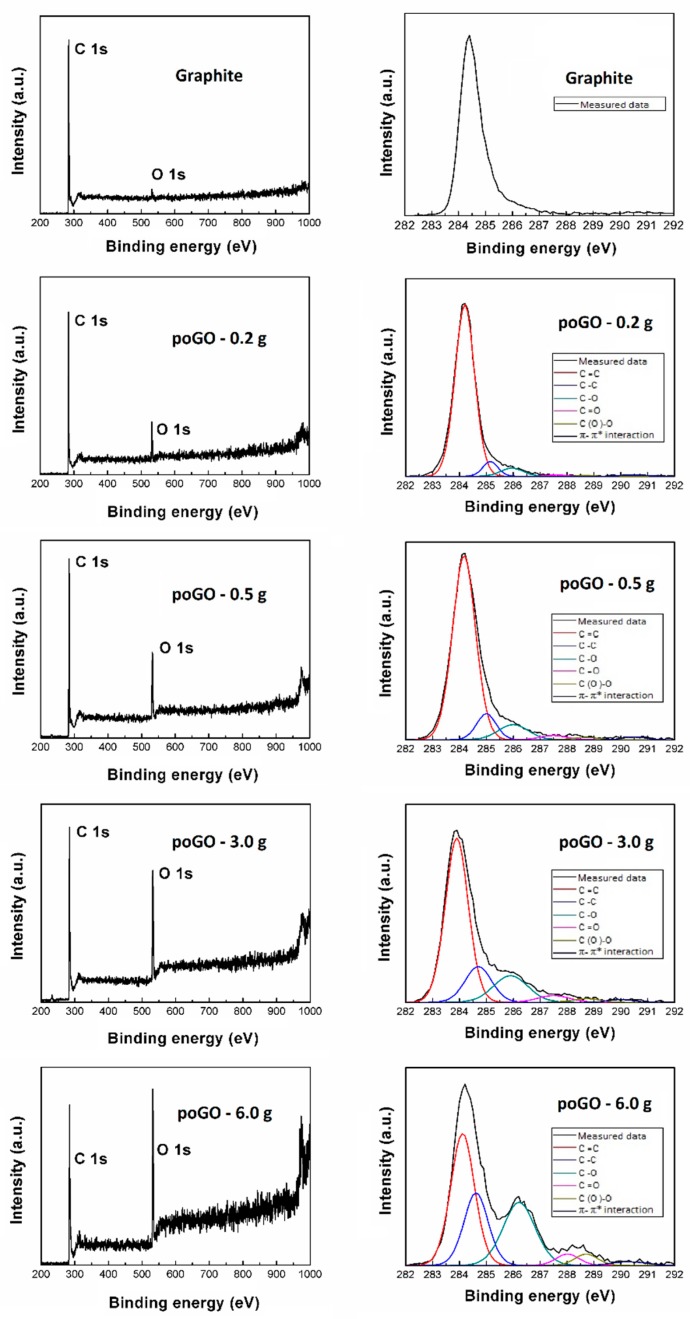
XPS spectra of poGOs: survey spectra (**left**) and C1s details (**right**).

Samples were also analyzed by XPS. For the XPS survey spectra, C1s peak was visible at ~284.5 eV and O 1s was found at ~532.5 eV (see Figure 2). The obtained C/O ratios are shown in Table 1. It is evident that C/O ratios measured through different methods cannot be the same, due to the different principles of these techniques. XPS is a surface sensitive method. Hence, the oxygen content is slightly higher, due to the fact that the oxidation took place mainly on the graphite surface. Nevertheless, the trend of increasing C/O ratio with the increasing amount of oxidizing agent used is significant. When more than 3.0 g was added, the oxidation started also inside the grains, hence the C/O ratio obtained by XPS for poGO-3.0 g and poGO-6.0 g is similar. A small amount of sulfur was detected in all samples. The sulfur was present in the form of sulfate ions.

XPS was used to calculate the amount of individual oxygen-containing functional groups of the samples (see Figure 2). The following carbon bonding states were identified in the graphite oxide: C=C (284.4 eV), C–C/C–H (285.2 eV), C–O (286.2 eV), C=O (287.8 eV), O–C=O (289.0 eV), and π–π* interactions (291.0 eV). The number of oxygen-containing functional groups was calculated by the deconvolution of C1s peak. The results (see Table 2) clearly document the trend of the increasing number of oxygen functionalities. It is also evident that oxygen was present predominantly in the form of hydroxyl groups in the less oxidized samples of poGO. 

**Figure 3:**
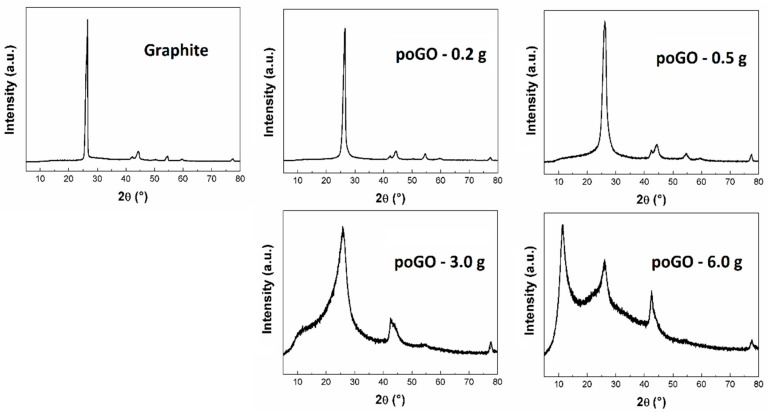
XRD measurements of poGOs.

The structure of partially-oxidized graphite oxides was investigated by X-Ray powder diffraction analysis (see Figure 3). While for graphite the strongest reflection (002) was at 26.5°, for fully oxidized graphite oxide, reflection at 10.1° was observed [34]. Although the interlayer distances in poGO-0.2 g, poGO-0.5 g, and poGO-3.0 g did not change a lot, in the case of poGO–6.0 g, the structural change was significant due to a higher oxidation level (see Table 3). Using the Debye–Scherrer method, the average particle sizes were calculated. The decrease in the average particle sizes with the increasing oxidation level of GO was caused by several factors: partial exfoliation and changes in the structure.

A sorption analyzer was used to determine surface area. For the pristine unoxidized graphite, a surface area of 7.0 m^2^ g^−1^ was obtained. The less oxidized samples, poGO-0.2 g and poGO-0.5 g, revealed slightly higher values of surface areas—7.6 m^2^ g^−1^ and 9.5 m^2^ g^−1^, respectively. A significant increase was observed in the case of the poGO-3.0 g sample. The surface area reached 15.3 m^2^ g^−1^ in this sample, while for the most oxidized sample, poGO-6.0 g, a value of 16.5 m^2^ g^−1^ was achieved. The measurements strongly support the results from XRD.

**Figure 4:**
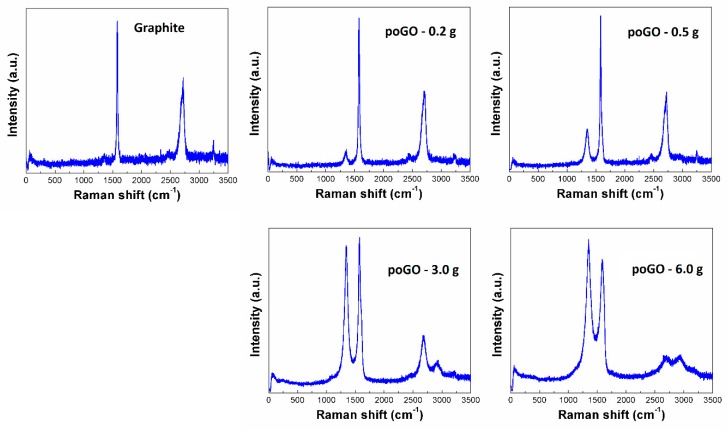
Raman spectra of poGOs.

In order to obtain information about the structure of the poGOs, Raman spectroscopy was measured (see Figure 4). The major band (so called G-band, at around 1580 cm^−1^) has been observed in all graphite-like structures [45,46]. It represents sp^2^ bonded carbon atoms in the non-defected aromatic rings. The 2D band is also present in all graphite-like structures at 2700 cm^−1^. This band is associated with the stacking of individual layers. Another observed band in partially-oxidized graphite oxides is called the D-band, and it is localized at around 1350 cm^−1^ [47,48]. The D-band indicates defects in the graphene layer, which are mostly associated with sp^3^ hybridization of carbon atoms (mostly due to the presence of oxygen functionalities). With an increase in the amount of KMnO_4_ used, the intensity of the D-band increases, while the intensity of the 2D band is suppressed. To consider the level of oxidation of the samples, the comparison of intensities (I_D_/I_G_) is crucial, since this value is directly proportional to the level of defects in the sample. The obtained I_D_/I_G_ ratios for all samples were between 0.10 for poGO-0.2 g and 1.17 for poGO-6.0 g. During the oxidation, the graphene layer with sp^2^ hybridized carbon atoms was impaired, and mainly hydroxyl functional groups were formed (sp^3^ hybridization) on the surface of individual layers, which led to increasing values of I_D_/I_G_. Also, epoxides can be formed on the graphene sheets, however, these functional groups can decompose after a few weeks or on exposure by daylight [49]. No luminescence was detected for all studied samples, in comparison to fully-oxidized graphite oxide samples [34]. 

**Figure 5:**
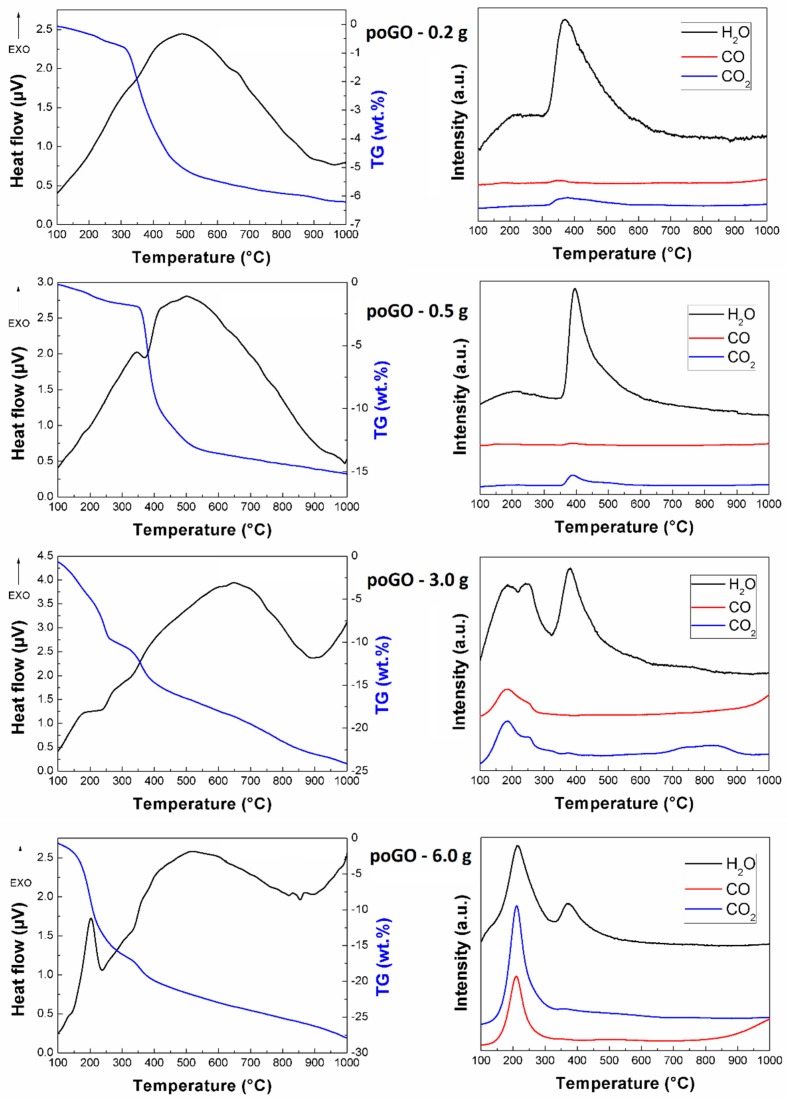
Thermal analysis of poGOs: heat flow and weight loss (**left**) and relative intensity of released gases (**right**).

Thermal stability was measured for all samples in an inert atmosphere. The heating of the samples was associated with weight loss, due to the evolution of gas molecules, such as carbon dioxide, carbon monoxide, and water (see Figure 5). Let us note that during a fast heating, other molecules can be released, too [50,51]. According to the observed data, the total weight loss of the prepared samples was proportional to the increasing level of oxidation. Also, the temperatures of major gas release were decreasing with the oxidation level. The samples poGO-0.2 g and poGO-0.5 g started to decompose at ~350 °C, whereas for poGO-3.0 g of poGO-6.0 g this occurred at lower temperatures. The exfoliation (typical for graphite oxides) was detected for the sample poGO-6.0 g at ~200 °C. The exfoliation was obviously caused by an extreme increase in interlayer pressure between the individual sheets.

**Figure 6:**
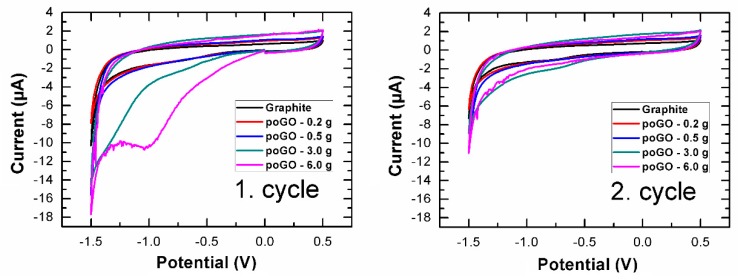
Measurements of inherent electrochemistry in first cycle (**left**) and second cycle (**right**).

Cyclic voltammetry provided information about the amount of electrochemically reducible oxygen-containing functionalities. According to the literature, peroxide, aldehyde, epoxide, and carboxyl functionalities are reduced at potentials 0.7, −1.0, −1.5, and −2.0 V versus the Ag/AgCl standard electrode, respectively. [52]. The samples poGO-0.2 g and poGO-0.5 g did not differ significantly from pristine graphite (see Figure 6). By contrast, a reduction in peroxide groups was observed in the samples poGO-3.0 g and poGO-6.0 g. The obtained results are in a good agreement with other analytic methods. The reduction occurred only in the first cycle, and no reverse oxidation was observed. During the second cycle, the number of oxygen functionalities (that were reduced) was significantly lower. 

## 4. Conclusions

In this paper, samples of partially-oxidized graphite oxide (poGO) were prepared by graphite oxidation. For the oxidation, different amounts of potassium permanganate were used. The prepared poGOs were subsequently analyzed in order to determine the oxidation level. The chemical composition of the samples was studied by EA, XPS, and EDS. It was found that the oxygen content increased proportionally to the amount of oxidizing agent. The interlayer distances in poGOs also increased, as well as the surface area of the poGOs. The obtained results were supported by STA-MS, where weight loss during the heating increased with the level of oxidation. Let us note that the results are only applicable to graphite of this particle size, they can vary significantly with starting material of different particle sizes. The results of our research can be useful for the fast, safe, and cost-effective synthesis of partially-oxidized graphite oxides. By oxidation using potassium permanganates we are able to tune the chemical composition. Samples with exact chemical composition can be used in various composite materials, e.g., in conductive polymers for wearable electronics (PEDOT/GO composites), for water-treatment (membranes or filters), or in building materials (self-cleaning surfaces). In addition, partially-oxidized graphite oxides with higher oxygen content can be used for the synthesis of thermally reduced graphene or as reactive precursors for further chemical modifications. 

## Figures and Tables

**Table 1:** Calculated C/O ratios of the partially-oxidized graphite oxides (poGOs). Measurements were obtained by EA, EDS, and XPS. Expected relative uncertainty is less than 1% for all analytic techniques.

Sample	C/O (EA)	C/O (EDS)	C/O (XPS)
PoGO-0.2 g	12.0	12.5	7.3
poGO-0.5 g	8.7	9.1	5.5
poGO-3.0 g	3.7	3.3	3.9
poGO-6.0 g	3.1	2.5	3.8

**Table 2:** Calculated number of individual oxygen functionalities in poGOs measured with XPS in at.%.

Sample	C=C	C–C	C–O	C=O	C(O)–O
poGO-0.2 g	87.3	6.1	5.1	0.9	0.6
poGO-0.5 g	77.9	9.8	8.6	2.4	1.3
poGO-3.0 g	61.8	15.4	14.9	5.0	2.9
poGO-6.0 g	41.5	23.2	26.7	3.5	5.1

**Table 3:** XRD measurements of (002) reflection of poGOs, interlayer distances, and average size of particles in poGOs, calculated by the Bragg equation and Debye–Scherrer method.

Sample	Diffraction Angle (°)	Interlayer Distance (Å)	Average Particle Size (Å)
poGO-0.2 g	26.48	3.36	94.61
poGO-0.5 g	26.14	3.41	43.59
poGO-3.0 g	25.76	3.46	12.43
poGO-6.0 g	11.35	7.79	-

## Supplementary Materials

The following are available online at https://www.mdpi.com/1996-1944/12/15/2367/s1, details in analytic methods.
