# Supplementary material for: Synthesis, Composition, and Properties of Partially Oxidized Graphite Oxides
Source: Materials (Basel). 2019 Jul 25;12(15):2367. doi: 10.3390/ma12152367 (PMC6695847; doi:10.3390/ma12152367)
Supplement: Supplementary file 1 [file materials-12-02367-s001.pdf]

materials

Supplementary Materials: Synthesis, Composition, and Properties of Partially Oxidized Graphite Oxides

Michal Lojka, Boris Lochman, Ondřej Jankovský, Adéla Jiříčková, Zdeněk Sofer and David Sedmidubský

Instruments and Settings

The morphology was investigated using scanning electron microscopy (SEM) with a FEG electron source (Tescan Lyra dual beam microscope). Elemental composition was determined using an energy dispersive spectroscopy (EDS) analyzer (X-Max^N) with a 20 mm^2 SDD detector (Oxford Instruments) and AZtecEnergy software. The samples were placed on a carbon conductive tape before the measurement. SEM and SEM-EDS measurements were carried out with a 10 kV electron beam.

X-Ray powder diffraction (XRD) was carried out at room temperature on Bruker D8 Discoverer powder diffractometer with parafocussing Bragg–Brentano geometry by using CuK$_\alpha$ radiation (λ = 0.15418 nm, U = 40 kV, I = 40 mA). Data were scanned over the angular range 5°–80° (2Θ). Interlayer distances and particle size were calculated in PANalytical's X'Pert High Score software.

Combustible elemental analysis (EA) was performed using a PE 2400 Series II CHNS/O Analyzer (Perkin Elmer, USA). The instrument was used in CHN operating mode to convert the sample elements to simple gases (CO_2, H_2O and N_2). The PE 2400 Analyzer automatically performed combustion, reduction, homogenization of product gases, separation and detection. An MX5 microbalance (Mettler Toledo) was used for precise weighing of the samples (1.5–2.5 mg per sample). The internal calibration was performed with N-phenyl urea.

Raman spectroscopy was performed with InVia Raman microscope (Renishaw, England) in backscattering geometry with CCD detector. DPSS laser (532 nm, 50 mW) with the applied power of 5 mW and 50× magnification objective was used for the measurement. Instrument calibration was achieved with a silicon reference which gives a peak position at 520 cm^{-1} and a resolution of less than 1 cm^{-1}. The samples were suspended in deionized water (1 mg/mL) and ultrasonicated for 10 minutes. The suspension was deposited on a small piece of silicon wafer and dried.

High-resolution X-Ray photoelectron spectroscopy (XPS) was performed with an ESCAProbeP spectrometer (Omicron Nanotechnology Ltd., Germany) with a monochromatic aluminum X-ray radiation source (1486.7 eV). The sample was applied on a conductive carbon tape before the analysis. Wide-scan surveys of all elements were performed, with subsequent high-resolution scans of the C 1s and O 1s.

The surface area was measured using a sorption analyzer, Coulter SA 3100 (Backman Coulter). The samples were outgassed for 4 h at 95 °C under high vacuum (VALUE) prior to the sorption experiments. The reason for such a low temperature is to avoid degradation and further decomposition of oxygen functionalities. A TCD nitrogen cooled (77 K) detector was used for the evaluation of the results using BET (Brunauer, Emmett and Teller) and Kelvin equations.

The measurement of graphite oxide exfoliation in inert atmosphere was performed by simultaneous thermal analysis (STA) using Setsys Evolution apparatus from Setaram. The evolved gases were analysed by OmniStar™ mass spectrometer from Pffeifer Vacuum. The measurement was performed in a dynamic helium atmosphere (50 mL.min^{-1}) using a heating rate of 10 °C.min^{-1}

The electrochemical characterization by means of cyclic voltammetry was performed using a potentiostat PGSTAT 204 (Metroohm Autolab B.V., The Netherlands). All glassy carbon electrodes were cleaned by polishing with an alumina suspension to renew the electrode surface then washed and wiped dry prior to any use. The samples were dispersed in DMF as the organic solvent to obtain a1 mg/mL suspension. The suspension was then sonicated for 5 minutes at room temperature before

every use. A cleaned GC electrode was then modified by coating with a 1.5 μL aliquot of the suspension and left to dry at ambient temperature to give a layer of randomly dispersed material on the GC surface. The modified GC electrodes, saturated Ag/AgCl reference electrode, and platinum counter electrode were then placed into an electrochemical cell which contains the electrolyte solution, and the data were then recorded. The electrolytes used were 50 mM, pH 7.2 phosphate buffer solution (PBS) as the blank buffer electrolyte and 10 mM potassium ferrocyanide dissolved in PBS. All measurements were performed for three consecutive scans at a scan rate of 100 mV/s.

© 2019 by the authors. Licensee MDPI, Basel, Switzerland. This article is an open access article distributed under the terms and conditions of the Creative Commons Attribution (CC BY) license (http://creativecommons.org/licenses/by/4.0/).
